# Evaluation of a multi-herb supplement for erectile dysfunction: a randomized double-blind, placebo-controlled study

**DOI:** 10.1186/1472-6882-12-155

**Published:** 2012-09-15

**Authors:** Gaurang R Shah, Manojkumar V Chaudhari, Suresh B Patankar, Shrikant V Pensalwar, Vilas P Sabale, Navneet A Sonawane

**Affiliations:** 1 Jivdaya Hospital, Dharmoday bldg, Jivdaya lane, L.B.S. Marg, Ghatkopar (West), Mumbai, 400 086, India; 2 Bhaghirathi Medical Foundation, 169, Parvati gaon, Pune, 400 009, India; 3 Institute of Urology, 32/2A, Erandwane, Gulwani Maharaj Road, Pune, 400 009, India; 4 Balaji Clinic, Devi Pada, Main Road, Borivali (East), Mumbai, 400 066, India; 5 Sabale Multispeciality Clinic, First floor, Vithal Acrade, Bhosari, Pune, 411 039, India; 6 Clinical Operations, Vedic Lifesciences Pvt. Ltd., B-118, Morya House, Link Road, Andheri (West), Mumbai, 400 053, India

## Abstract

**Background:**

Evidence is lacking for multi-ingredient herbal supplements claiming therapeutic effect in sexual dysfunction in men. We examined the safety and efficacy of VigRX Plus (VXP) – a proprietary polyherbal preparation for improving male sexual function, in a double blind, randomized placebo-controlled, parallel groups, multi-centre study.

**Methods:**

78 men aged 25–50 years of age; suffering from mild to moderate erectile dysfunction (ED), participated in this study. Subjects were randomized to receive VXP or placebo at a dose of two capsules twice daily for 12 weeks. The international index of erectile function (IIEF) was the primary outcome measure of efficacy. Other efficacy measures were: Erectile Dysfunction Inventory of Treatment Satisfaction (EDITS), Serum testosterone, Semen analysis, Investigator’s Global assessment and Subjects’ opinion.

**Results:**

In subjects receiving VXP, the IIEF-Erectile Function (EF) scores improved significantly as compared to placebo. After 12 weeks of treatment, the mean (sd) IIEF-EF score at baseline increased from 16.08 (2.87) to 25.08 (4.56) in the VXP group versus 15.86 (3.24) to 16.47 (4.25) in the placebo group (P < 0.0001). Similar results were observed in each of the remaining four domains of the IIEF (orgasmic function, sexual desire, intercourse satisfaction, and overall satisfaction).There was a significant difference for VXP versus placebo comparison of mean (sd) EDITS scores of patients: 82.31(20.23) vs 36.78(22.53) and partners :(82.75(9.8) vs 18.50(9.44);*P* < 0.001. Thirty-five out of 39 (90%) subjects from the VXP group and one (3%) from the placebo group wished to continue with the treatment they received. Investigator’s global assessment rated VXP therapy as very good to excellent in more than 50% patients and placebo therapy as fair to good in about 25% of patients. Incidence of side effects and subject’s rating for tolerability of treatment was similar in both groups.

**Conclusions:**

VigRX Plus was well tolerated and more effective than placebo in improving sexual function in men.

**Trial Registration:**

Clinical Trial Registry India, CTRI/2009/091/000099, 31-03-2009

## Background

Erectile dysfunction (ED) or impotence is defined as the persistent inability to attain and maintain an erection sufficient to permit satisfactory sexual performance [1].

One of the oldest diseases known to mankind, ED affects 52% of 40- to 70-year-old men [2] with estimates predicting the incidence to rise to over 320 million by the year 2025 [3]. The earliest reports of medical therapies for ED are found in ancient medical literature prescribing the use of numerous herbs and natural ingredients for sexual rejuvenation and a healthy progeny. As sexual medicine evolved, introduction of intracavernous injection therapy followed by phosphodiesterase type-5 (PDE type-5) inhibitors revolutionized the treatment of ED. Despite the enormous success of pharmacological agents and a wide variety of treatment choices currently available, the ED sufferer continues to resort to natural alternatives or herbal supplements to regain his sexual vigor.

Clinicians on the other hand, do not wholeheartedly recommend herbal or alternative therapies, mainly due to a lack of adequate evidence from robustly designed scientific studies [4-6]. In the absence of any regulatory obligations to undertake rigorous testing for safety and efficacy of dietary supplements [7], there is no impetus for evaluation of herbal or dietary supplements before they are sold over-the-counter. Manufacturers base the advertising or labeling claims for such products on the testing of individual ingredients rather than the whole composition [8]. There are also risks attendant upon self-medication and unmonitored use of these products [9]. Evidence of contamination of herbal products with PDE type-5 inhibitors [10] further prompts the need for companies to act responsibly and encourage third-party scientists to conduct efficacy and safety studies on natural products claiming therapeutic health benefits.

In the present study, we evaluated the safety and efficacy of a multi-herb formulation marketed as VigRx Plus (Leading Edge Herbals), created for enhancement of sexual health in men. Development of this product was based on the preliminary evaluation of a first generation product, VigRX, consisting of a proprietary blend of *Panax ginsengSerenoa repensGingko bilobaCrataegus laevigataPtychopetalum olacoidesErythroxylum catuabaCuscuta chinensis*, and *Epimedium sagittatum* extract. In two different studies in male Sprague–Dawley rats, VigRX was shown to engender a marked improvement in sexual behaviour including decreased intromission and ejaculation latencies, and increased intromission, ejaculation and mounting frequencies [11]. In the same study, assays for pharmaceutical adulteration found that VigRX did not contain any detectable levels of known PDE-5 inhibitors including sildenafil, tadalafil, vardenafil or related analogues. In *vitro* assays also determined that VigRX is able to inhibit the Rho-kinase. Rho-kinase is an enzyme that plays an important role in maintaining the flaccid state of the penis through cavernosal vasoconstriction [12] and is being increasingly considered as emerging target for the treatment of erectile dysfunction [13]. VigRX, however, exhibited a relatively high inhibition concentration in the Rho-kinase inhibition assay, indicating that a large dose would be necessary to achieve similar results in a living system. Hence, three more ingredients *Tribulus terrestrisTurnera diffusa* and Bioperine® (piperine) were added to the formulation. The resulting new formulation, named VigRX Plus (Table 1), was evaluated for its aphrodisiac properties in male albino Wistar rats. Treatment with VigRX Plus at the dose of 450 mg/kg showed a significant increase in ejaculation frequency on day 7 and a non-significant increase on day 14 with a marginal increase in testosterone concentration in serum and number of spermatogonia cells in seminiferous tubules of testes *(Unpublished data;Available uponrequest).* An acute oral toxicity study of VigRX Plus tablet blend observed no lethality, nor adverse effects at single oral doses up to 4,000 mg/kg in female rats *(Unpublished data,Available uponrequest)*.

**Table 1:** Composition of VigRX Plus

**Ingredients (Botanical names)**	**Part used**	**Quantity per capsule (mg)**
Panax ginseng	Root	100
Serenoa repens	Berry	100
Crategus rivularis	Berry	100
Ginkgo Biloba	Leaf	100
Turnera diffusa	Leaf	100
Tribulus terrestris	Vine	075
Erythroxylum catuaba	Bark	050
Ptychopetalum olacoides	Bark	050
Cuscuta chinensis	Seed	025
Epimedium sagittatum	Leaf	015
Bioperine (extract from Piper nigrum fruit)	-	005
**Total amount**		720

With the accrued preclinical evidence, VigRx Plus demonstrated potential as a novel agent for management of ED. It was thus imperative to evaluate its safety and efficacy in humans.

## Methods

### Administration

The study was registered at the Clinical Trials Registry India (Registration No: CTRI/2009/091/000099, 31-03-2009) and received approval from an independent ethics committee (IEC) of Noble Hospital, and AMAI Charitable Trust, Pune, India. The study was conducted at outpatient clinics of participating urologists and general physicians, from May 2009 to December 2009. The trial conduct was monitored to ensure compliance to the ethical principles of Declaration of Helsinki, International Conference on Harmonisation (ICH) - Good Clinical Practice (GCP) guidelines and IEC approved protocol. Independent quality assurance auditors verified the quality of the data generated from the study.

### Participants

Men aged 25–50 years, seeking treatment for sexual dysfunction, at the outpatient clinics of investigators, were offered participation in this study. The volunteers gave written informed consent before being assessed on the IIEF scale. Those with a score of 11–23 on the EF domain of the IIEF were eligible. Major illnesses (including diabetes and cardiovascular diseases) and sexual dysfunction due to anatomical, surgical or pharmacological causes were excluded.

### Randomisation and blinding

Subjects were randomized to receive VXP or placebo at a dose of 2 capsules twice daily (each capsule containing 360 mg of active or placebo composition) for 12 weeks. The randomization sequence was generated manually, in blocks of four, by drawing chits of paper from a bag, by a person not involved in the execution of the study or the analysis of its results. Investigator, patient and statistician were blinded to the random assignment. Randomization sequence was concealed in tamper-evident envelopes maintained in the custody of investigators. Envelope integrity was checked at each monitoring visit to ensure concealment of random allocation.

The study medications were indistinguishable in terms of appearance, weight and taste. Both active and placebo were packed in identical containers with identical labels carrying patient ID and treatment week as distinctive markers for dispensing and monitoring compliance. Use of any other substance or product for treatment of male sexual dysfunction was prohibited during the study. Prior use of conventional or alternative medicine required a wash out of 7 and 15 days respectively.

### Outcome measures

The IIEF questionnaire was used to evaluate the treatment effect on the sexual functioning in subjects of this study. The questionnaire described by Rosen *et al.* is a self-reported, validated instrument for measuring erectile dysfunction and monitoring response to treatment [14]. It evaluates several aspects of sexual function over five important domains: Erectile Function (EF), Sexual Desire (SD), Orgasmic Function (OF), Intercourse satisfaction (IS), and Overall satisfaction (OS). The IIEF was administered at baseline and 4-week intervals.

Treatment satisfaction of subjects and their female partners was assessed through responses to the Erectile Dysfunction Inventory of Treatment Satisfaction (EDITS) questionnaire. Seminal parameters and serum testosterone levels were assessed at baseline and end of study. Safety was evaluated through incidence of adverse events, changes in laboratory parameters and subject’s rating for tolerability of treatment. Subjects were also asked to declare whether they wished to continue with the trial medication. Additionally, at study end, investigators rated response to therapy as excellent, good, fair or poor.

### Statistical analysis

Analysis for safety was done on an intent-to-treat (ITT) population of subjects who received at least one dose and had at least a single post-baseline measurement. Efficacy analysis was done on a per protocol (PP) data set consisting of subjects completing all protocol-required visits. Statistical analyses were performed using SAS® for windows (version 9.2; SAS Institute, Cary, NC, USA), True Epistat version 5.0; and MS Excel XP. Mean changes in IIEF-EF, IS,OF,SD and OS domains, and total IIEF scores from baseline to end of treatment were evaluated by analysis of covariance (ANCOVA) with baseline scores as the covariate. Data on EDITS (patient and partner versions), seminal parameters and serum testosterone were analyzed by independent sample *t* test. Chi-square test was used to analyze investigators’ global assessment and subject’s opinion. All statistical tests were applied at a 5% level of significance.

## Results

### Patient disposition and baseline characteristics

A total of 78 subjects were recruited into the study. While all men receiving VXP completed the study duration of 12 weeks, one in the placebo group was withdrawn due to his unwillingness to continue participation, and two others were lost to follow-up (Table 1). Baseline characteristics of the participants including severity of sexual dysfunction were comparable across the two groups (Table 2). Almost all patients in the VXP (38/39) and placebo (36/36) group were finding it difficult to extremely difficult maintaining their erection to completion of intercourse. In both groups, attempts to intercourse ranged from 3–4 or5-6 in the four weeks prior to entering the study.

**Figure 1:**
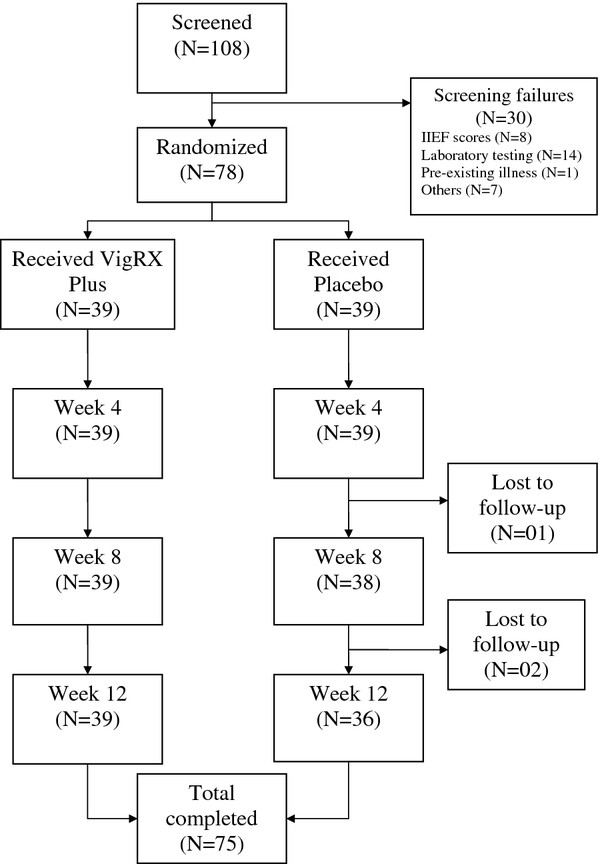
Flow of participants.

**Table 2:** Baseline characteristics

**Characteristics**	**VigRX Plus**	**Placebo**
	**(N = 39)**	**(N = 39)**
Age in years		
Mean (SD)	35.23 (6.62)	34.33 (5.89)
Range	25-48	25-44
Duration (years)		
Mean (SD)	2.27 (1.80)	2.01(1.35)
Range	0.25-10	0.25-6
Mild to moderate^†^ ED (n)	21	23
Moderate ED (n)	18	16

ED, erectile dysfunction; IIEF-EF, international index of erectile function-erectile function.

^†^Mild-to-moderate ED and moderate ED were defined as IIEF-EF:17–21 and IIEF scores 11–16 respectively.

### Efficacy

#### Primary efficacy parameters

IIEF-EF domain

Treatment with VXP showed a statistically significant (*P* < 0.0001) increase of IIEF–EF domain scores from baseline to end of study (12 weeks) as compared to placebo (Table 3). Mean (sd) increase in EF score was of 9 (4.95) points in the VXP group and 0.61 (2.43) points in the placebo group. In 13/ 39 (34%) patients in the VXP group and one (3%) in the placebo group, the erections were *almost alwaysor always* hard enough for penetration (Q2 IIEF).The ability to penetrate the partner (Q3 IIEF) and maintain erection after penetration (Q4 IIEF) increased by 59% and 63% in subjects receiving VXP and by 4% and 9% in those receiving placebo, respectively. Almost all subjects receiving VXP, and two receiving placebo rated their confidence to get and keep erection as *high tovery high*. 14 subjects in the VXP group and one in the placebo group achieved >25 (no dysfunction) scores at the end of study.

**Table 3:** Effect of VigRX Plus on the IIEF domains

***IIEF Domains***	***VigRX Plus***				***Placebo***			
	**Baseline**	**EoT**	**Change**	**95%CI**	**Baseline**	**EoT**	**Change**	**95%CI**
EF,Q1-5,15	16.08 (2.87)	25.08 (4.56)*	9 (4.95)	7.40,10.60	15.86 (3.24)	16.47 (4.25)	0.61(2.43)	−0.21,1.43
Q1	3.38 (0.67)	4.64 (0.67)	1.26 (0.88)	0.98,1.54	3.33 (0.53)	3.38 (0.77)	0.06 (0.58)	−0.14,0.25
Q2	2.71(0.65)	4.17 (0.82)	1.46 (0.94)	1.16,1.76	2.66 (0.63)	2.80 (0.75)	0.14 (0.42)	−0.0,0.28
Q3	2.69 (0.57)	4.12 (0.80)	1.43 (0.85)	1.15,1.70	2.63 (0.59)	2.75 (0.69)	0.11(0.46)	−0.05,0.26
Q4	2.56 (0.50)	4.02 (0.81)	1.46 (0.91)	1.16, 1.76	2.44 (0.56)	2.66 (0.68)	0.22 (0.48)	0.06, 0.38
Q5	2.23 (0.78)	3.94 (0.89)	1.71 (1.02)	1.38, 2.04	2.27 (0.85)	2.33 (0.89)	0.06 (0.47)	−0.10, 0.21
Q15	2.48 (0.56)	4.15 (0.87)	1.66 (1.03)	1.33, 1.99	2.5 (0.56)	2.52 (0.84)	0.03 (0.56)	−0.16, 0.21
IS, Q6-8	7.28 (1.70)	11.58 (2.46)*	4.3 (2.47)	3.50,5.10	7.13 (1.76)	7.72(1.98)	0.58 (1.44)	0.09,1.07
OF, Q9-10	7.84 (0.93)9(	9.23 (1.13)*	1.38 (1.24)	0.98,1.78	7.77 (1.20)	7.77 (1.53)	0.0 (0.92)	−0.31,0.31
SD,Q11-12	6.35 (1.11)	9.05 (1.36)*	2.69 (1.73)	2.13,3.25	6.47 (1.28)	6.41 (1.5)	−0.05 (1.62)	−0.60,0.50
OS, Q13-14	5.46 (1.10)	8.17 (1.73)*	2.71 (1.86)	2.10,3.31	5.61 (0.96)	5.47 (1.50)	−0.14 (0.90)	−0.45,0.16
Total IIEF,Q1-15	42.56 (5.09)	63.13 (10.06)*	20.10 (11.08)	16.51,23.69	42.54 (5.10)	43.86 (8.45)	1.0 (5.73)	−0.93,2.93

IIEF scores are expressed as Mean (SD), *P < 0.001 is statistically significant for change from baseline as compared to placebo.CI, confidence intervals; EF, erectile function; EoT, end of treatment; IS, intercourse satisfaction; OF, orgasmic function; OS, overall satisfaction; SD, sexual desire.

IIEF- Other domains

Following 12 weeks of treatment, there was a significant improvement from baseline in all other IIEF domains (SD, OF, IS and OS) in the VXP group as compared to placebo (Table 3). Percentage increase in each of the domains was greater with VXP therapy than with placebo (Table 2).Greatest increases were observed in the erectile function and intercourse satisfaction domains.

**Figure 2:**
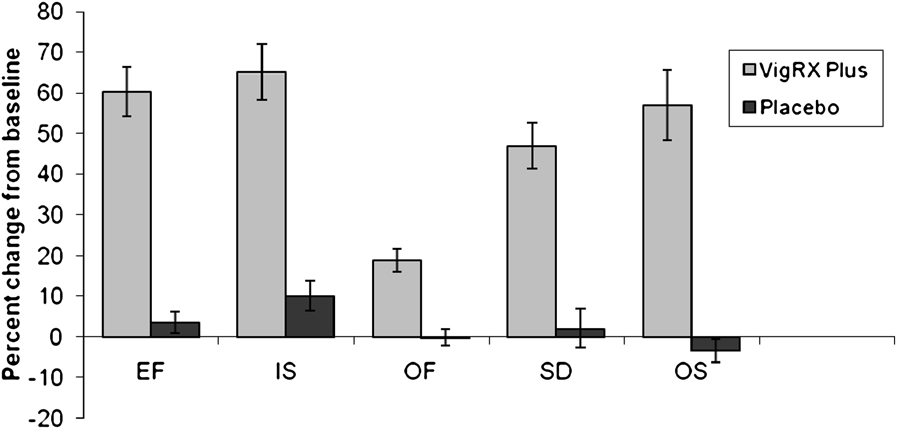
** Percentage change from baseline in IIEF domains.** Treatment with VigRX Plus significantly improved all important domains of sexual functioning in men. Note that an increase in the ability to achieve and maintain an erection (erectile function) was accompanied by increases in intercourse and overall satisfaction. *P < 0.001 for% change (mean ± sem) from baseline to end-of-treatment with VigRX Plus as compared to placebo.

Frequency of intercourse attempts increased in both groups with 15 patients in the VXP group and 10 in the placebo group making 11+ attempts in the last four weeks of the study. However, a majority of patients in the VXP group said that their sexual intercourse was *highly tovery highlyenjoyable* whereas for most patients in the placebo group sexual intercourse was either *fairly enjoyableor notenjoyable*.

Sexual desire was frequently rated as *high tovery high* in the VXP group, and *moderate tohigh* in patients receiving placebo. More patients in the VXP than in the placebo group were *very satisfied* with their overall sex life sexual relationship with their partner. The mean (sd) increase in total IIEF was 20.10(11.08) in the VXP group and 1.0(5.73) in the placebo group (*P* < 0.001).

Overall, the significant rise in every domain of the IIEF indicates that VXP had improved various aspects of sexual functioning in men.

#### Secondary efficacy parameters

Subjects and female partners in the VXP group reported significantly higher (*P* < 0.0001) scores of treatment satisfaction by EDITS than those in the placebo group (Table 4). The fact that both patient and partners were highly satisfied is a reflection of the improved quality of sexual life after treatment with VXP.

**Table 4:** Effect on EDITS scores

	***VigRX Plus***	**Placebo**
Patient	82.31 (20.23)	36.78 (22.53)
	(N = 39)	(N = 36)
Partner	88.75 (9.80)	18.50 (9.44)
	(N = 12)	(N = 10)

EDITS scores are expressed as Mean (SD).

There was no significant difference in the changes in sperm count, semen volume and sperm motility between the two treatment groups. Serum Testosterone levels were not altered significantly in any of the study groups (Table 5). VXP received greater number of favorable (p < 0.001) responses in investigator’s global assessment of efficacy, as compared to placebo. VXP therapy was rated as excellent in 8, very good in 18 and good in 11 subjects. In a majority of subjects (26 out of 36) in the placebo group, efficacy was rated as poor. Thirty-five out of 39 (90%) subjects (*P* < 0.0001) in the VXP group answered ‘*yes*’ to the question, ‘*Would youtake thesame productin thefuture ifyou sufferfrom thesame condition?*’ Only one subject (3%) from the placebo group responded positively to this question.

**Table 5:** Effect on seminal parameters and serum testosterone

***Variable***	***VigRX Plus***		**Placebo**	
	**Baseline**	**12 weeks**	**Baseline**	**12 weeks**
Sperm count (million/ml)	49.45 (27.85)	47.15 (25.31)	58.29 (30.02)	63.97 (20.83)
	(N = 22)	(N = 22)	(N = 18)	(N = 18)
Semen volume (ml)	1.75 (0.63)	2.11 (0.53)	1.97 (0.70)	2.22 (0.88)
	(N = 22)	(N = 22)	(N = 18)	(N = 18)
Serum testosterone (ng/Dl)	544.46 (207.64)	527.66 (155.47)	518.10 (197.51)	471.75 (160.38)
	(N = 37)	(N = 37)	(N = 25)	(N = 25)

Data are expressed as Mean (SD).

### Safety

VXP was generally well tolerated in this study. Out of a total of 23 adverse events occurring in the study, 11 were reported from the VXP group and 12 from the placebo group. The most common (7/23) adverse event was fever of mild severity, with the incidence of the event being similar in both. A single serious adverse event occurred in this study when one subject from VXP group was hospitalized due to malarial infestation and subsequently withdrawn from the study. No significant difference was observed in the tolerability of treatment as a majority of subjects in both groups (31 in VXP and 28 in placebo) rated the tolerability as very good.

## Discussion

Over recent years, the use of complementary and alternative medicines has become increasingly popular [15], and ED is one condition for which herbal supplements are heavily promoted and easily accessible [16]. ED sufferers often seek alternatives, since many are reticent to express their sexual problems to physicians [17], or are dissatisfied with current therapies. Even after restoration of erectile function, successful treatments have nevertheless been abandoned for such reasons as fear of possible side effects, aversion to drug-dependent erections, high drug cost, dislike of need to plan sexual activity, and lack of sexual interest [18].

The present study evaluated VigRX Plus, a poly-herbal supplement purported to offer natural sexual enhancement in men. The efficacy results of this first-in-human study were generally consistent with the effects demonstrated by VXP in animal experiments. VXP was found to be effective in improving the erectile function in men with sexual dysfunction. Statistically significant increases in IIEF scores showed that VXP had a therapeutic benefit that was superior to placebo. From the improvement displayed in all five domains of the IIEF (erection quality, sexual desire, orgasm quality, intercourse satisfaction and overall satisfaction), it appears that VXP may help in enhancing the overall quality of sexual experience in men.

### Multi-herb combination: synergistic efficacy or compromised safety?

#### Efficacy

Often, multi-herb supplements are formulated with the aim of achieving a net additive or synergistic effect of individual ingredients with similar clinical or pharmacological actions. Practitioners of traditional medicine believe that combinations of herbs improve efficacy and reduce adverse effects [19]. But whether such combinations synergize or even simulate the therapeutic action of their components remains largely unknown.

Among the herbal constituents of VXP, only Korean red ginseng has been previously evaluated on the IIEF scale, in two double-blind, randomized, controlled studies. Of the two, a similar study in 60 patients with mild to moderate ED showed an increase in the IIEF-five item scores from a baseline of 16.4 ± 2.9 to 21.0 ± 6.3, after receiving 1000 mg (3 times daily) of ginseng for 12 weeks [20]. The increase of 9 points in IIEF- EF with VXP in our study is comparable to the approximately 5-point increase in the ginseng study. In the other 45-patient study designed as cross-over, mean increases in IIEF scores in the Ginseng (900 mg. 3 times daily) group were significantly higher than in those who received placebo (baseline 28.0 +/− 16.7 and 38.1 +/− 16.6 versus 30.9 +/− 15.7, p <0.01) [21]. In response to the global efficacy question, 60% of the patients answered that Korean red ginseng improved erection, which is in agreement with the 72% (26 out of 36) patients global response observed in our study.

The aphrodisiac properties of *tribulus* were demonstrated in animal models [22-24] and in a human study [25] where *tribulus* extract (3x250 mg/day for 3 weeks) increased the sexual drive in 60% of diabetic and non-diabetic ED men. This was accompanied by a significant improvement in the levels of dehydroepiandrosterone-sulphate (DHEA) - a hormone necessary for the maturation process of spermatozoa; however no significant differences were observed in testosterone.

*Serenoa repens* (Saw Palmetto) in an open study for longer than 48 weeks in 155 men with clinically diagnosed BPH, prostate symptoms and quality of life improved significantly from baseline over the 2 years while sexual function improved in the second year [26]. *Turnera diffusa* (Damiana)’s sexually invigorating properties were evidenced from studies in sexually exhausted male rats [27,28]. Gingko biloba has been shown to relax vascular smooth muscle and this mechanism of action is thought to contribute to an improvement in ED [29]. Icariin, the active ingredient from *Epimedium sagitattum*, improved the erectile function and expression of nitric oxide synthase in castrated rats [30,31].

Thus, overall, the VXP multiple-herb preparation appears to have exhibited the properties of its individual ingredients. But whether the blend was more potent than single herbs is unlikely to be ascertained from this study, mainly due to different study methodologies and populations of individual herb studies, causing difficulty in comparison of results.

#### Safety

Safety deserves no less attention than efficacy, while choosing an appropriate therapy for ED. Negative consequences associated with treatment, have been identified as important to patients receiving treatment for ED [32]. VXP was apparently safe and well-tolerated in this study. This is consistent with the long history of safe use of herbs present in VXP. Contrary to the general belief, recent reports have questioned the safety of some herbs including *Ginkgo biloba* which has concerns over blood-thinning effects [33] and ocular toxicity [34]. That none of these adverse effects were reported for VXP is assuring; however, caution should be exercised while interpreting the safety results as they are not determined over the long-term.

### ED: Through the patient’s perspective

Unlike many other medical disorders, ED is a condition for which nobody other than the patient can decide upon a treatment option or interpret its success [35]. Both patient and partner satisfaction are considered to be relevant to understanding and predicting treatment continuation in ED [32,36].The growing importance of patient choice in clinical decision-making is evident from several trials that have attempted to evaluate patient preference or treatment-switching patterns amongst available PDE-5 inhibitors: the first-line ED treatment. Long-term adherence to PDE-5 inhibitors seems to be low despite their high efficacy, good tolerability, and ease of administration [37].

Our study primarily focused on obtaining the patients’ perspective on treatment success, mainly by employing patient-reported measures like the IIEF and EDITS. We found that improvements in sexual function recorded by IIEF were corroborated by patient and partner satisfaction, as evidenced by significantly greater EDITS (patient and partner) scores reported for VXP as compared to placebo. Additionally, subjects’ opinions and investigators’ rating were also in favor of VXP.

### Limitations and future action

First of all, the study sought to examine the effects of VigRx Plus in young men who had no apparent co-morbid conditions. ED on the other hand, is recognized as a disorder of older adults suffering from systemic diseases such as diabetes mellitus, cardiovascular disease and hypertension [38,39]. The seemingly atypical patient characteristics limit the extrapolation of the clinical benefit of VXP to that in the presence of concurrent diseases and medications that are commonly seen in a larger ED population of older adults.

However, young patients reporting erectile difficulties are not unknown of. Heruti *et al.* reported that at least one out of three young men could be suffering from ED, emphasizing that this condition is a major health concern of the young as well [40]. Another study has observed that young men with ED have comparatively more difficulty in adjusting to life influenced by less relationship satisfaction, greater depressive symptomatology, more negative reaction from partners and less job satisfaction [41]. Moreover, unlike the ‘asexual older men’ who perceive ED as a normal and irreversible part of the ageing process [42], young men may find ED a serious impediment to an active and perfect sexual life, and are hence likely to seek help more often than their older counterparts.

Secondly, the study does not gather enough evidence to conclude whether the ED was of organic, psychogenic or mixed origin. Even in the absence of any detectable underlying condition or risk factor, an organic aetiology cannot be refuted as ED is known to share common endothelial dysfunction pathways with many vascular diseases [43] and manifests 2–3 years beforehand [44] to serve as an early warning of such diseases, particularly in men 45 years or younger [45]. Experts therefore recommend that physicians consider a man with ED and no cardiac symptoms as a cardiac or vascular patient until proved otherwise [46]. Thus, we hypothesize that vascular and other conditions though noticeably absent could have existed in a latent or subclinical phase when patients entered the study. Only long- term follow-up programs can help verify if ED had actually served as a predictor of vascular abnormalities in these patients.

Then again, we did not specifically rule out psychogenic ED by the use of psychometric assessments or by evaluating the intactness of sleep-time erections through nocturnal penile tumescence and rigidity testing. Given that psychogenic ED should be confirmed only after ascertaining the presence of psychosocial factors as the predominant or exclusive cause [47], a definitive statement that the patients in our study were mainly suffering from psychogenic ED would remain unjustified. Future studies with VXP should aim at differentiating between various forms of ED in order to acquire a better understanding of the intervention in treating ED of diverse aetiology.

An intriguing finding of the study was the small sized placebo effect observed for patient reported outcomes. It is a clear departure from the usually observed placebo response rate of 25% (1 out of every 4 men benefits) in ED trials [48]. Here again, the finding suggests a preponderance of organic ED and also a tendency for non-response towards placebo. The fact that patients had a history of over two years of erectile difficulties before enrolling themselves for the study indicates that they were not treatment naive and had exhausted their placebo response to a new treatment option .The learning that occurs after experiencing the benefits and side effects (or the lack thereof) from a previous medication may have shaped patients’ expectations about a therapeutic effect, allowing them to decipher whether or not they received the active treatment during the study. This partly explains why patient responses in the placebo group were poor while those in the active group were comparable to that of pharmacological drugs for ED. However, considering the small sized placebo effect observed in this study, the results described should be viewed with caution. It may have benefited the treatment results as significantly better, even if minimal. In clinical trials of drug treatments, placebo responses have often been substantial, usually significantly and statistically better than no treatment (baseline).

Patient expectations could also be influenced by foreknowledge of the treatment assignment. Differences in color or size of pills can serve as cues in differentiating the active treatment from placebo to introduce a response bias. However, this possibility is eliminated from our study as the indistinguishable treatments and strict implementation of operational measures ensured adequate concealment of allocation sequence and prevented any lapses in the integrity of study blinding.

To summarize, results obtained from evaluation in young men with no overt organic causes may offer limited generalizability, nevertheless, represent an important subset of ED patients who are potentially amenable to therapy and at the same time are least likely to exhibit a placebo response that can confound the determination of active treatment effects. The study has set grounds for further evaluation of VXP in patients with well-recognized risk factors and distinctly identified forms of ED.

## Conclusions

VigRX Plus was well-tolerated and effective in improving erectile function in men. Confirmation of the beneficial effect in a broader ED population is recommended.

## Abbreviations

EF, Erectile function; IS, Intercourse satisfaction; OF, Orgasmic function; OSD, Overall satisfaction; SD, Sexual desire.

## Competing interests

Authors GRS, MVC, SBP, SVP and VPS were compensated for their participation as study planners, organizers, investigators, and writers. NAS is an employee of an organization that received funds for monitoring the study. The funders had no influence on the scientific integrity of the preparation for research, the study execution, and the preparation of the manuscript for publication.

## Authors' contributions

All authors made substantial contributions to study conception, acquisition of data, and preparation of the manuscript. All authors read and approved the final manuscript.

## Authors’ information

GRS: M.S., M.Ch (Urology), is a consulting urologist at leading hospitals in Mumbai, and a member of the Mumbai Urology Society. Areas of special interest include male infertility and impotence. MVC: M.D., PhD, is an associate professor and practitioner of herbal medicine, Ashtang Ayurved college and hospital, Pune. SBP: M.S., M.Ch (Urology) is Professor and Head of Department at B.J.Medical College, Pune; chief urologist, Institute of Urology; and a member of Urology society of India. SBP was an investigator for clinical trials of PDE5 inhibitors in sexual dysfunction; and is the author of several international peer-reviewed publications in urology. SVP: MBBS, is a general medical practitioner in Mumbai and has been actively involved in clinical trials of herbal medications. VPS: M.Ch. (Urology) is a practicing urologist at Pune. NAS: B.A.M.S., is Manager, clinical operations, Vediclifesciences Pvt. Ltd. Mumbai; has executed numerous phase II to III clinical trials; and is also a practitioner of herbal medicine.

## Pre-publication history

The pre-publication history for this paper can be accessed here:

http://www.biomedcentral.com/1472-6882/12/155/prepub
